# Manoalide Preferentially Provides Antiproliferation of Oral Cancer Cells by Oxidative Stress-Mediated Apoptosis and DNA Damage

**DOI:** 10.3390/cancers11091303

**Published:** 2019-09-04

**Authors:** Hui-Ru Wang, Jen-Yang Tang, Yen-Yun Wang, Ammad Ahmad Farooqi, Ching-Yu Yen, Shyng-Shiou F. Yuan, Hurng-Wern Huang, Hsueh-Wei Chang

**Affiliations:** 1 Institute of Biomedical Science, National Sun Yat-sen University, Kaohsiung 80424, Taiwan; 2 Department of Radiation Oncology, Faculty of Medicine, College of Medicine, Kaohsiung Medical University, Kaohsiung 80708, Taiwan; 3 Department of Radiation Oncology, Kaohsiung Medical University Hospital, Kaohsiung 80708, Taiwan; 4 Cancer Center, Kaohsiung Medical University Hospital, Kaohsiung Medical University, Kaohsiung 80708, Taiwan (Y.-Y.W.) (S.-S.F.Y.); 5 School of Dentistry, College of Dental Medicine, Kaohsiung Medical University, Kaohsiung 80708, Taiwan; 6 Center for Cancer Research, Kaohsiung Medical University, Kaohsiung 80708, Taiwan; 7 Department of Molecular Oncology, Institute of Biomedical and Genetic Engineering (IBGE), Islamabad 54000, Pakistan; 8 Department of Oral and Maxillofacial Surgery Chi-Mei Medical Center, Tainan 71004, Taiwan; 9 Translational Research Center, Kaohsiung Medical University Hospital, Kaohsiung 80708, Taiwan; 10 Department of Medical Research, Kaohsiung Medical University Hospital, Kaohsiung 80708, Taiwan; 11 Institute of Medical Science and Technology, National Sun Yat-sen University, Kaohsiung 80424, Taiwan; 12 Department of Biomedical Science and Environmental Biology, Kaohsiung Medical University, Kaohsiung 80708, Taiwan

**Keywords:** marine sponge, natural product, anticancer drug, oral cancer inhibition

## Abstract

Marine sponge-derived manoalide has a potent anti-inflammatory effect, but its potential application as an anti-cancer drug has not yet been extensively investigated. The purpose of this study is to evaluate the antiproliferative effects of manoalide on oral cancer cells. MTS assay at 24 h showed that manoalide inhibited the proliferation of six types of oral cancer cell lines (SCC9, HSC3, OC2, OECM-1, Ca9-22, and CAL 27) but did not affect the proliferation of normal oral cell line (human gingival fibroblasts (HGF-1)). Manoalide also inhibits the ATP production from 3D sphere formation of Ca9-22 and CAL 27 cells. Mechanically, manoalide induces subG1 accumulation in oral cancer cells. Manoalide also induces more annexin V expression in oral cancer Ca9-22 and CAL 27 cells than that of HGF-1 cells. Manoalide induces activation of caspase 3 (Cas 3), which is a hallmark of apoptosis in oral cancer cells, Ca9-22 and CAL 27. Inhibitors of Cas 8 and Cas 9 suppress manoalide-induced Cas 3 activation. Manoalide induces higher reactive oxygen species (ROS) productions in Ca9-22 and CAL 27 cells than in HGF-1 cells. This oxidative stress induction by manoalide is further supported by mitochondrial superoxide (MitoSOX) production and mitochondrial membrane potential (MitoMP) destruction in oral cancer cells. Subsequently, manoalide-induced oxidative stress leads to DNA damages, such as γH2AX and 8-oxo-2’-deoxyguanosine (8-oxodG), in oral cancer cells. Effects, such as enhanced antiproliferation, apoptosis, oxidative stress, and DNA damage, in manoalide-treated oral cancer cells were suppressed by inhibitors of oxidative stress or apoptosis, or both, such as *N*-acetylcysteine (NAC) and Z-VAD-FMK (Z-VAD). Moreover, mitochondria-targeted superoxide inhibitor MitoTEMPO suppresses manoalide-induced MitoSOX generation and γH2AX/8-oxodG DNA damages. This study validates the preferential antiproliferation effect of manoalide and explores the oxidative stress-dependent mechanisms in anti-oral cancer treatment.

## 1. Introduction

Oral cancer is one of the high incidence cancers worldwide [1], especially in Southeast Asia and Taiwan. Betel quid chewing, smoking, and alcohol consumption are high risk factors for oral cancer [2]. Oral cancer causes serious morbidity and mortality [3]. Current therapies for oral cancer patients include surgery or chemoradiation, or both. However, chemoradiation commonly shows severe side effects in oral cancer patients [4]. Therefore, continuous drug screening and development for oral cancer therapy remains a challenge.

Marine natural products provide an abundant resource for development of anti-cancer agents [5,6,7,8]. Besides corals, marine sponges provide diverse sources of natural products from the ocean. Marine sponges are marine resources with a wide range of bioactive compounds and secondary metabolites with potential therapeutic effects [9,10,11]. Bioactive compounds of marine sponges and their microbial consortia are known for their anticancer, anti-inflammatory, antiviral, and antibiotic effects [11,12].

In 1980, manoalide, an antibiotic sesterterpenoid isolated from the marine sponge *Luffariella variabilis*, was discovered [13]. In 1999, manoalide was reported to function as an analgesic and anti-inflammatory agent [14]. This anti-inflammatory effect may be caused by the inhibition of phospholipase A2 (PLA2) by manoalide [14]. Moreover, manoalide also functions as inhibitors for phospholipase C (PLC) [15,16] and calcium channels [17]. Manoalide reached Phase II (antipsoriatic) clinical trial, although it was discontinued due to formulation problems [18].

In addition to anti-inflammatory effects, the anti-cancer effects of manoalide have not been extensively studied. For example, manoalide showed a cytotoxic effect against murine lymphoma LI210 and human epidermoid carcinoma KB cells [19]. However, the anticancer effect against oral cancer cells was not studied as yet.

Natural products, such as marine sponges, commonly showed antioxidant properties [20,21]. Some marine sponge-derived natural products showed both cytotoxic and antioxidant activities [22,23,24]. Manoalide inhibits superoxide production in colon cancer cells (HT29-D4) [25], suggesting that manoalide may have an antioxidant potential. Interestingly, antioxidants possess double-edge sword activities to regulate cellular reactive oxygen species (ROS). For example, antioxidants at physiological concentrations may decrease ROS and benefit cell health but induce ROS that damage cells at high concentrations [26]. Hence, the ROS modulating effect of manoalide on oral cancer cells warrants further investigation. Furthermore, drugs with inducing ROS generation ability may preferentially kill cancer cells but show little damage to normal cells [27]. Whether manoalide causes a preferential killing to oral cancer cells needs further to be examined.

In this study, we hypothesized that manoalide may preferentially inhibit the proliferation of oral cancer cells. To examine this hypothesis, the preferential antiproliferation effect of manoalide on oral cancer cells was studied by analyzing cell survival, cell cycle, apoptosis, oxidative stress, and DNA damage.

## 2. Results

### 2.1. Cell Viability of Manoalide-Treated Oral Cancer and Normal Oral Cells with or Without Pretreatments of NAC or Z-VAD

**Figure 1:**
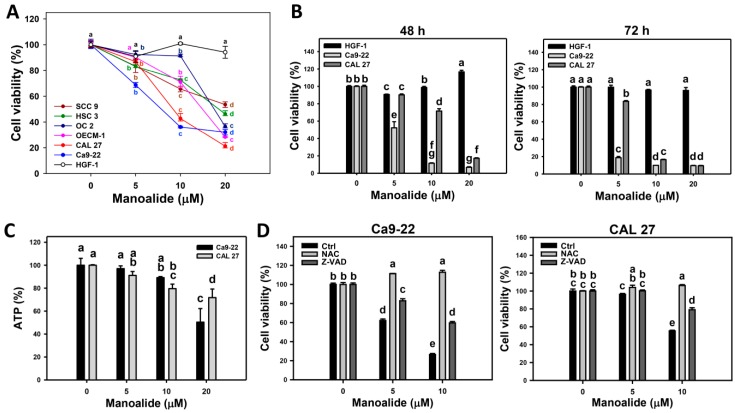
Cell viabilities of oral cancer cells after manoalide treatment and its *N*-acetylcysteine (NAC)/apoptosis inhibitor Z-VAD-FMK (Z-VAD) effects. Cells were treated with 0, 5, 10, and 20 μM of manoalide. All treatments have the same concentration of DMSO. (**A**) MTS assay-based cell viabilities for 24 h. Oral cancer (CAL 27, Ca9-22, OECM-1, OC-2, HSC 3, and SCC9) cells and oral normal (HGF-1) cells were included. (**B**) MTS assay-based cell viabilities for 48 and 72 h for oral cancer (CAL 27 and Ca9-22) and oral normal (HGF-1) cells. (**C**) Statistical of 3D spheroid formation for manoalide-treated oral cancer (Ca9-22 and CAL 27) cells for 72 h. (**D**) NAC and Z-VAD effects on MTS viability of manoalide-treated oral cancer cells. Pretreatment conditions were 8 mM, 1 h for NAC and 100 μM, 2 h for Z-VAD. Following pretreatment or not, oral cancer (Ca9-22 and CAL 27) cells were post-incubated with 5 and 10 μM manoalide for 24 h. Data, means ± SDs (*n* = 3). Data were analyzed by one-way ANOVA with Tukey HSD Post Hoc Test. Data showing the same small lettersrepresent nonsignificant differences whereas data showing no overlapping same small letters are significant difference (*p* < 0.05–0.001).

Cell viability was determined by mitochondrial enzyme activity-based MTS assay. Figure 1A shows that manoalide dose-responsively decreases the viability (%) of oral cancer cells (CAL 27, Ca9-22, HSC3, OECM-1, SCC9, and OC-2), but it only slightly decreases oral normal cells (human gingival fibroblasts (HGF-1)), i.e., their IC_50_ values of manoalide are 7.8, 9.1, 14.9, 17.4, and 18.5 μM at 24 h MTS assay. Among the oral cancer cells, Ca9-22 and CAL 27 cells belong to different oral locations (gingival and tongue) and show higher cytotoxicity upon manoalide treatment. Accordingly, Ca9-22 and CAL 27 cells were selected for the following assays to investigate the detailed mechanisms of anti-oral cancer cells by manoalide. Figure 1B shows that 48 and 72 h treatments of manoalide dose-responsively decrease the viability (%) of oral cancer cells, but it only slightly decreases oral normal cells (HGF-1), i.e., the IC_50_ values of manoalide-treated oral cancer Ca9-22 and CAL 27 cells are 5.3 versus 14.0 μM and 3.1 versus 7.5 μM at 48 and 72 h MTS assay, respectively. Furthermore, the photo images of 3D sphere formation pattern of oral cancer cells are provided (Figure S1A). Its cell viability needs to be determined by ATP detection. As shown in Figure 1C, the ATP-detected 3D sphere formation ability of oral cancer cells (Ca9-22 and CAL 27) was decreased by manoalide treatment.

To address the role of oxidative stress and apoptosis in cell viability, the ROS scavenger *N*-acetylcysteine (NAC) [28,29] and apoptosis inhibitor Z-VAD-FMK (Z-VAD) [30] were used. The cell morphologies were abnormal in manoalide-treated oral cancer (Ca9-22 and CAL 27) cells, especially at higher concentrations (Figure S1B). However, these manoalide-induced abnormal changes on morphologies were recovered by NAC pretreatment and partly recovered by Z-VAD pretreatment (Figure S1B). Moreover, manoalide-suppressed cell viabilities in oral cancer cells were completely inhibited by a NAC pretreatment and partly inhibited by a Z-VAD pretreatment (Figure 1D).

### 2.2. Cell Cycle Changes of Manoalide-Treated Oral Cancer Cells with or Without Pretreatments of NAC or Z-VAD

**Figure 2:**
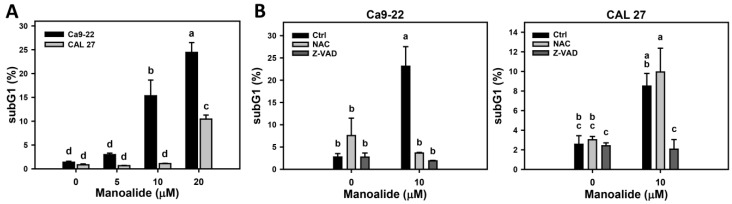
Cell cycle changes of manoalide-treated oral cancer (Ca9-22 and CAL 27) cells. (**A**) Statistical results of the subG1 (%) for manoalide-treated oral cancer cells in Figure S2A. Cells were treated with 0, 5, 10, and 20 μM of manoalide for 24 h. (**B**) Statistical result of the subG1 (%) for NAC, Z-VAD, and/or manoalide-treated oral cancer cells in Figure S2B. Cells were pretreated with 8 mM, 1 h for NAC or 100 μM and 2 h for Z-VAD, and they were then post-incubated with 10 μM of manoalide for 24 h. Data, means ± SDs (*n* = 3). Data were analyzed by one-way ANOVA with Tukey HSD Post Hoc Test. Data showing no overlapping same small letters represent significant difference (*p* < 0.05–0.001).

7-Aminoactinomycin D (7AAD) is a DNA staining dye for measuring the different cell cycle phases. Figure S2A shows the pattern changes of cell cycle progression for oral cancer cells (Ca9-22 and CAL 27) after manoalide treatment. The subG1 and > 4N populations appear at 10 and 20 μM of manoalide for Ca9-22 cells and at 20 μM for CAL 27 cells. Figure 2A shows that the subG1 populations are increased after manoalide treatment.

To address the role of oxidative stress and apoptosis in cell cycle distribution, the NAC and Z-VAD were used. Figure S2B shows the effect of NAC and Z-VAD pretreatments on pattern of cell cycle progression for manoalide-treated oral cancer cells and shows cell cycle disturbances (subG1 and > 4N populations). Figure 2B shows these manoalide-induced subG1 accumulations were recovered by NAC pretreatment and partly recovered by Z-VAD pretreatment.

### 2.3. Apoptosis of Manoalide-Treated Oral Cancer Cells with or Without Pretreatments of NAC or Z-VAD

**Figure 3:**
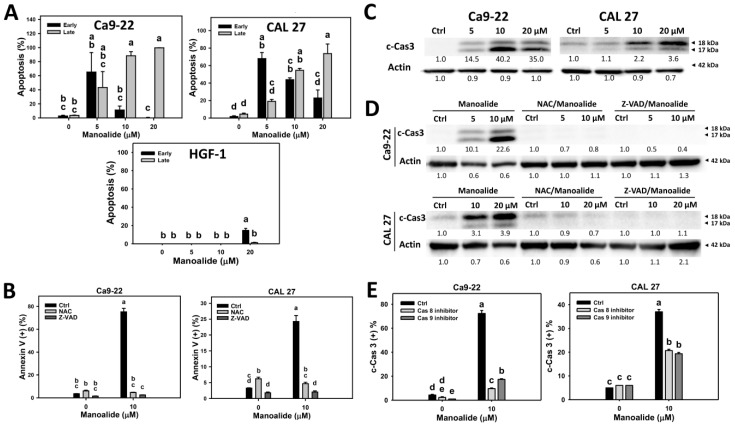
Apoptosis changes in manoalide-treated oral cancer (Ca9-22 and CAL 27) cells and normal oral (HGF-1) cells. (**A**) Statistical results of the annexin V/7AAD method in manoalide-treated oral cancer cells and normal oral (HGF-1) cells in Figure S3A. Cells were treated with different concentrations of manoalide for 24 h. Early and late apoptosis were, respectively, counted by the populations in the annexin V (+)/7AAD (−) and annexin V (+)/7AAD (+) regions, i.e., Q3 and Q2. (**B**) Statistics results of annexin V/7AAD method in NAC, Z-VAD, and/or manoalide-treated oral cells in Figure S3B. Cells were pretreated with NAC (8 mM, 1 h) or Z-VAD (100 μM, 2 h), and posttreated with manoalide (10 μM, 24 h). Apoptosis was represented by the sum of early and late apoptosis, i.e., annexin V (+)/7AAD (+ or −). (**C**) Western blotting for detecting apoptosis in manoalide-treated oral cancer cells. (**D**) Western blotting for detecting apoptosis in NAC, Z-VAD, and/or manoalide-treated oral cells. Cleaved forms caspase 3 (c-Cas 3) were used to detect apoptosis. Actin was the internal control. (**E**) Statistical results of c-Cas 3 positive levels in Cas 8 inhibitor, Cas 9 inhibitor, and/or manoalide-treated oral cells in Figure S6. Cells were pretreated with Cas 8 inhibitor Z-IETD-FMK (100 μM, 2 h) or Cas 9 inhibitor Z-LEHD-FMK (100 μM, 2 h), and posttreated with manoalide (10 μM, 24 h). Data were analyzed by one-way ANOVA with Tukey HSD Post Hoc Test. Data, means ± SDs (*n* = 3). Data showing no overlapping same small letters represent significant difference (*p* < 0.05–0.001).

Apoptosis was detected by the annexin V/7AAD method. Figure S3A shows that the populations of oral cancer (Ca9-22 and CAL 27) cells shift from annexin V (−)/7ADD (−) to annexin V (+)/7ADD (−) at 5 μM of manoalide and further shift to annexin V (+)/7ADD (+) at 10 and 20 μM. In contrast, normal oral cells (HGF-1) show only a slight shift to apoptosis region. Therefore, cell populations of oral cancer cells shift from alive, early apoptosis, to late apoptosis when the concentrations of manoalide increase. Figure 3A shows that manoalide mainly induces early apoptosis at 5 μM, moderately induces late apoptosis at 10 μM, and mainly induces late apoptosis at 20 μM in oral cancer cells. However, manoalide-treated HGF-1 cells induce little apoptosis, which is undetectable at 5 and 10 μM and is less than 15% for early apoptosis at 20 μM.

The involvement of oxidative stress in the apoptosis for manoalide-treated oral cancer cells were further examined (Figure S3B and Figure 3B). Figure S3B shows that the populations of manoalide-induced late apoptosis shift to early apoptosis or living status by NAC or Z-VAD pretreatment. Figure 3B shows that NAC or Z-VAD pretreatments decrease the manoalide-induced apoptosis for both oral cancer cells, Ca9-22 and CAL 27.

To further validate that apoptosis is induced by manoalide in oral cancer cells, western blotting analysis was performed. Figure 3C shows that manoalide induces overexpression of cleaved forms of caspase 3 (c-Cas 3) in both oral cancer cells, Ca9-22 and CAL 27. Figure S4A showing the procaspase 3 and c-Cas 3 patterns also supports this finding. Moreover, this manoalide-induced c-Cas 3 is suppressed by NAC or Z-VAD pretreatments in both oral cancer cells, Ca9-22 and CAL 27 (Figure 3D). All the raw data for western blotting are provided (Figures S4B and S5).

To identify the initiator caspase responsible for the activation of Cas-3, the involvements of Cas 8 and Cas 9 were examined by using its inhibitors [31]. Figure S6A shows that the immunofluorescence of c-Cas 8- and c-Cas 9-staining of manoalide-treated oral cancer cells is higher than the control. Figure S6B shows that the populations of manoalide-induced c-Cas 3 shift to a low level by pretreatments of Cas 8 or Cas 9 inhibitors. Figure 3E shows that Cas 8 or Cas 9 inhibitor pretreatments decrease the manoalide-induced c-Cas 3 activation for both oral cancer cells, Ca9-22 and CAL 27, suggesting that initiator caspase, such as both Cas 8 and Cas 9, are responsible for the c-Cas-3 activation in manoalide-treated oral cancer cells.

### 2.4. ROS Production of Manoalide-Treated Oral Cancer and Normal Oral Cells

**Figure 4:**
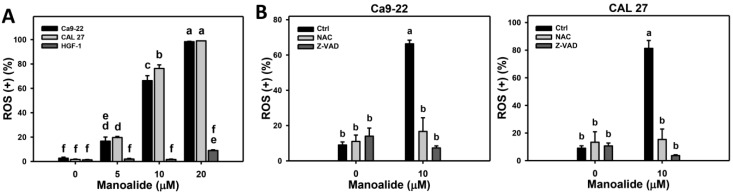
ROS changes in manoalide-treated oral cancer (Ca9-22 and CAL 27) and normal oral (HGF-1) cells. Cells were treated with different concentrations of manoalide for 10 min. (**A**). Statistical results of ROS (+) (%) for manoalide-treated oral cancer and oral normal cells in Figure S7A. (**B**) Statistical results in NAC, Z-VAD, and/or manoalide-treated oral cells in Figure S7B. Cells were pretreated with NAC (8 mM, 1 h) or Z-VAD (100 μM, 2 h), and posttreated with manoalide (10 μM, 10 min). Data were analyzed by one-way ANOVA with Tukey HSD Post Hoc Test. Data, means ± SDs (*n* = 3). Data showing no overlapping same small letters represent significant differences (*p* < 0.05–0.001).

2’,7’-dichlorodihydrofluorescein diacetate (DCFH-DA) can react with ROS to generate products for flow cytometry detection [32]. Figure S7A shows the ROS patterns of manoalide-treated oral cancer (Ca9-22 and CAL 27) and normal oral (HGF-1) cells. Figure 4A shows that the ROS productions of Ca9-22 and CAL 27 cells are dramatically induced when the concentrations of manoalide increase. In contrast, the ROS productions of HGF-1 cells stay unchanged at 5%, at less than 10 μM and slightly increased to 25% at 20 μM.

To address the role of oxidative stress and apoptosis in manoalide-induced ROS production, pretreatments of NAC and Z-VAD were used, and its ROS patterns were shown in Figure S7B. Figure 4B shows that the manoalide-induced ROS productions are inhibited by NAC and Z-VAD pretreatments for both oral cancer cells, Ca9-22 and CAL 27.

### 2.5. Mitochondrial Superoxide (MitoSOX) Production of Manoalide-Treated Oral Cancer Cells with or Without Pretreatment of MitoTEMPO

**Figure 5:**
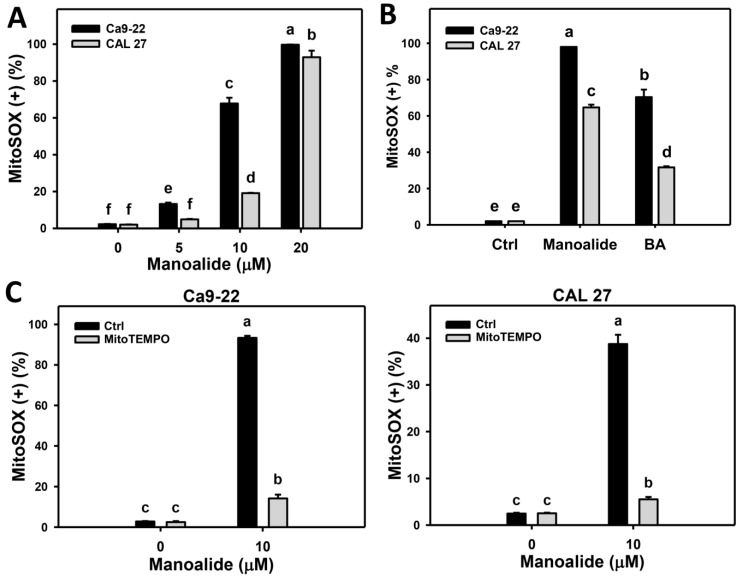
Change of mitochondrial superoxide (MitoSOX) production in manoalide-treated oral cancer (Ca9-22 and CAL 27) cells. (**A**) Statistical results of MitoSOX (+) (%) for manoalide-treated oral cancer cells in Figure S8A. Cells were treated with different concentrations of manoalide for 24 h. (**B**) Statistical results of positive control of MitoSOX (+) (%) for oral cancer cells in Figure S8B. Cells were treated with betulinic acid (BA; 25 μM, 24 h) as the positive control treatment for comparison to manoalide (10 μM, 24 h). (**C**) Statistical results of MitoSOX (+) (%) in MitoSOX inhibitor (MitoTEMPO) and/or manoalide-treated oral cells in Figure S8C. Cells were pretreated with MitoTEMPO (20 μM, 1 h) and posttreated with manoalide (10 μM, 24 h). Data were analyzed by one-way ANOVA with Tukey HSD Post Hoc Test. Data, means ± SDs (*n* = 3). Data showing no overlapping same small letters represent significant difference (*p* < 0.05–0.001).

MitoSOX™ Red can react with intra-mitochondrial superoxide to generate products for flow cytometry detection [33]. Figure S8A shows the populations of oral cancer (Ca9-22 and CAL 27) cells shift to MitoSOX (+) region when the concentrations of manoalide increase. Figure 5A shows that the MitoSOX production of Ca9-22 and CAL 27 cells dramatically increase. Figure S8B shows the positive control treatment (betulinic acid; BA) [34] for MitoSOX patterns in oral cancer. Figure 5B shows that the manoalide induces more MitoSOX productions than that of BA in Ca9-22 and CAL 27 cells. 

Moreover, the involvement of MitoSOX in manoalide-treated oral cancer cells was further validated using MitoSOX inhibitor (MitoTEMPO). Figure S8C shows the MitoSOX patterns of MitoTEMPO pretreatment effects against manoalide-treated oral cancer cells. Figure 5C shows that MitoTEMPO pretreatment decreases the manoalide-induced MitoSOX production for both oral cancer cells, Ca9-22 and CAL 27.

### 2.6. Membrane Potential (MitoMP) of Manoalide-Treated Oral Cancer Cells with or Without Pretreatments of NAC or Z-VAD

**Figure 6:**
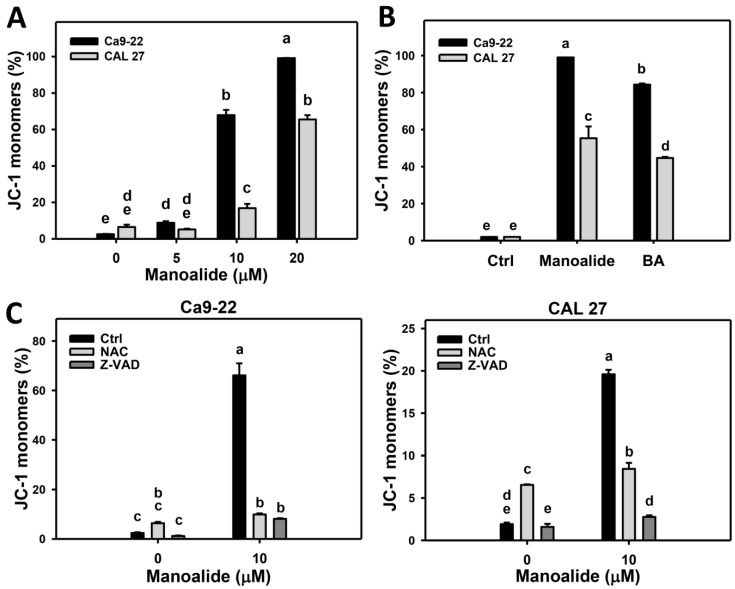
Change of membrane potential (MitoMP) in manoalide-treated oral cancer (Ca9-22 and CAL 27) cells. (**A**) Statistical results of JC-1 monomers (%) for manoalide-treated oral cancer cells in Figure S9A. Cells were treated with different concentrations of manoalide for 24 h. High JC-1 monomers (%) indicates low MitoMP, i.e., the MitoMP depolarization. (**B**) Statistical results of positive control of low MitoMP for oral cancer cells in Figure S9B. Cells were treated with betulinic acid (BA; 25 μM, 24 h) as the positive control treatment for comparison to manoalide (10 μM, 24 h). (**C**) Statistical results of JC-1 monomers (%) for NAC, Z-VAD, and/or manoalide-treated oral cells in Figure S9C. Cells were pretreated with NAC (8 mM, 1 h) or Z-VAD (100 μM, 2 h) and posttreated with manoalide (10 μM, 24 h). Data, means ± SDs (*n* = 3). Data were analyzed by one-way ANOVA with Tukey HSD Post Hoc Test. Data showing no overlapping same small letters represent significant difference (*p* < 0.05–0.001).

JC-1 aggregate (red fluorescent) form can concentrate at mitochondria, and JC-1 monomer form (green fluorescent) escape from mitochondria, reflecting the intact and depolarized MitoMP, respectively [35]. These fluorescent signals were detected by flow cytometry. Figure S9A shows that the populations of oral cancer (Ca9-22 and CAL 27) cells shift from JC-1 aggregates MitoMP (+) to JC-1 monomers MitoMP (−) region when the concentrations of manoalide increase. Figure 6A shows that the JC-1 monomers MitoMP (−) population is dose-responsively increased in manoalide-treated oral cancer cells. Figure S9B shows the positive control treatment (betulinic acid; BA) for MitoMP patterns in oral cancer. Figure 6B shows that the manoalide induces more JC-1 monomers MitoMP (−) productions than that of BA in Ca9-22 and CAL 27 cells.

To address the role of oxidative stress and apoptosis in manoalide-suppressed MitoMP, pretreatments of NAC and Z-VAD were used and its MitoMP patterns are shown in Figure S9C. Figure 6C shows that the manoalide-induced JC-1 monomer generations are inhibited by NAC and Z-VAD pretreatments for both oral cancer cells, Ca9-22 and CAL 27.

### 2.7. Flow Cytometry-Based DNA Damage Changes of Manoalide-Treated Oral Cancer Cells with or Without Pretreatments of NAC or Z-VAD

**Figure 7:**
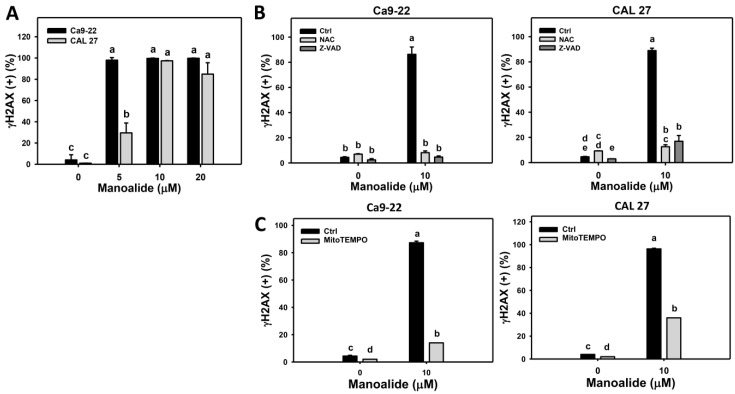
Change of γH2AX DNA damage in manoalide-treated oral cancer (Ca9-22 and CAL 27) cells. Cells were treated with the indicated concentrations of manoalide for 24 h. (**A**) Statistical results of γH2AX (+) (%) for manoalide-treated oral cancer cells in Figure S10A. (**B**) Statistical results of γH2AX (+) (%) in NAC, Z-VAD, and/or manoalide-treated oral cancer cells in Figure S10B. Cells were pretreated with 8 mM, 1 h for NAC or 100 μM, 2 h for Z-VAD, and then post-incubated with 10 μM of manoalide for 24 h. (**C**) Statistical results of γH2AX (+) (%) in MitoSOX inhibitor (MitoTEMPO) and/or manoalide-treated oral cancer cells in Figure S10C. Cells were pretreated with MitoTEMPO (20 μM, 1 h) and posttreated with manoalide (10 μM, 24 h). Data were analyzed by one-way ANOVA with Tukey HSD Post Hoc Test. Data, means ± SDs (*n* = 3). Data showing no overlapping same small letters represent significant difference (*p* < 0.05–0.001).

γH2AX is known as a DNA double strand break marker [36]. Figure S10A shows the populations of oral cancer (Ca9-22 and CAL 27) cells shift to γH2AX (+) region when the concentration of manoalide increases. Figure 7A shows that the γH2AX (+) population is dose-responsively increased in manoalide-treated oral cancer cells. To address the role of oxidative stress and apoptosis in manoalide-induced γH2AX, pretreatments of NAC and Z-VAD were used, and its γH2AX patterns are shown in Figure S10B. Figure 7B shows that the manoalide-induced γH2AX (+) (%) are inhibited by NAC and Z-VAD pretreatments for both oral cancer cells, Ca9-22 and CAL 27. Moreover, the involvement of MitoSOX for manoalide-induced γH2AX in oral cancer cells was further examined using MitoSOX inhibitor (MitoTEMPO). Figure S10C shows the γH2AX patterns of MitoTEMPO pretreatment effects against manoalide-treated oral cancer cells. Figure 7C shows that MitoTEMPO pretreatment decreases the manoalide-induced γH2AX (+) (%) for both oral cancer cells, Ca9-22 and CAL 27.

**Figure 8:**
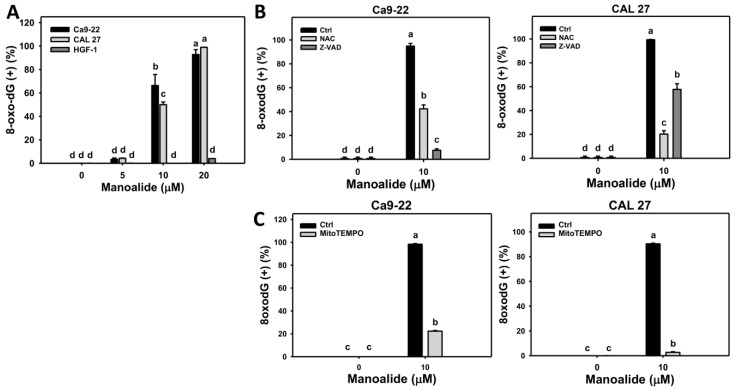
Change of 8-oxodG DNA damage in manoalide-treated oral cancer (Ca9-22 and CAL 27) and normal oral (HGF-1) cells. Cells were treated with the indicated concentrations of manoalide for 24 h. (**A**) Statistical results of 8-oxodG (+) (%) for manoalide-treated oral cancer cells in Figure S11A. (**B**) Statistical results of 8-oxodG (+) (%) in NAC, Z-VAD, and/or manoalide-treated oral cancer cells in Figure S11B. Cells were pretreated with 8 mM, 1 h for NAC or 100 μM, 2 h for Z-VAD, and then post-incubated with 10 μM of manoalide for 24 h. (**C**) Statistical results of 8-oxodG (+) (%) in MitoSOX inhibitor (MitoTEMPO) and/or manoalide-treated oral cancer cells in Figure S11C. Cells were pretreated with MitoTEMPO (20 μM, 1 h) and posttreated with manoalide (10 μM, 24 h). Data were analyzed by one-way ANOVA with Tukey HSD Post Hoc Test. Data, means ± SDs (*n* = 3). Data showing no overlapping same small letters represent significant difference (*p* < 0.05–0.001).

8-Oxo-2’-deoxyguanosine (8-oxodG) is one of the typical types of oxidative DNA damage [37]. Figure S11A shows the populations of oral cancer (Ca9-22 and CAL 27) cells shift to the 8-oxodG (+) region when the concentrations of manoalide increase, while the populations of 8-oxodG (+) in normal oral (HGF-1) cells are few. Figure 8A shows that the 8-oxodG (+) population is dose-responsively increased in manoalide-treated oral cancer cells; however, 8-oxodG (+) was rarely appeared in normal oral (HGF-1) cells. To address the role of oxidative stress and apoptosis in manoalide-induced 8-oxodG, pretreatments of NAC and Z-VAD were used and its 8-oxodG patterns are shown in Figure S11B. Figure 8B shows that the manoalide-induced 8-oxodG (+) (%) is inhibited by NAC and Z-VAD pretreatments for both oral cancer cells, Ca9-22 and CAL 27. Moreover, the involvement of MitoSOX for manoalide-induced 8-oxodG in oral cancer cells was further examined using MitoSOX inhibitor (MitoTEMPO). Figure S11C shows the 8-oxodG patterns of MitoTEMPO pretreatment effects against manoalide-treated oral cancer cells. Figure 8C shows that MitoTEMPO pretreatment decreases the manoalide-induced 8-oxodG (+) (%) for both oral cancer cells, Ca9-22 and CAL 27.

## 3. Discussion

The hypothesis that manoalide may preferentially inhibit the proliferation of oral cancer cells was validated in this study. In the following, we compare the manoalide sensitivity in different cancer cells and discuss the role of oxidative stress in preferential killing, apoptosis, and DNA damage in oral cancer cells.

### 3.1. Manoalide Sensitivity in Different Cancer Cells

Manoalide showed a cytotoxic effect against human epidermoid carcinoma KB cells (IC_50_ = 0.725 μM) [19] without providing information for treatment time and methods. Moreover, KB cells were reported as misidentified; these were HeLa cells, rather than oral cancer cells [38]. In the current study, oral cancer Ca9-22, CAL 27, OECM1, OC2, and HSC3 cells, respectively, show the IC_50_ values of manoalide with 7.8, 9.1, 14.9, 17.4, and 18.5 μM at 24 h MTS assay. It is noted that the drug safety for manoalide in normal cell lines was firstly demonstrated in normal oral cells (HGF-1), which remained healthy below 25 μM in a 24 h MTS assay. These results suggest that manoalide provides a preferentially killing effect to oral cancer cells and shows little damage to normal oral cells.

### 3.2. Manoalide Induced Oxidative Stress Contributes to Preferential Killing Against Oral Cancer Cells

Both 2,2-azinobis (3-ethyl-benzothiazoline-6-sulfonic acid) (ABTS) and hydroxyl scavenging activities of manoalide showed IC_50_ values for 14.3 and 18 μM (Figure S12), suggesting that manoalide causes the antioxidant abilities. Recently, antioxidants were reported to display dual concentration effects [26,39], i.e., low and high concentrations, respectively, decrease and increase intracellular ROS levels. Similarly, we found that manoalide differentially induced ROS production between oral cancer cells and normal oral cells, i.e., manoalide induces higher ROS production in oral cancer cells than in normal oral cells (Figure 4). Moreover, manoalide also induces other types of oxidative stresses, such as MitoSOX production and MitoMP depolarization (Figure 5 and Figure 6). Using NAC or MitoTEMPO pretreatments, oxidative stresses, such as ROS generation (Figure 4B), MitoSOX production (Figure 5C), and MitoMP depolarization (Figure 6C), as well as antiproliferation, were suppressed (Figure 1D). Therefore, the preferential killing effect of manoalide is oxidative stress-dependent in oral cancer cells. This finding also supports the rationale that ROS-modulating drugs provided preferential killing effects against several types of cancer cells [27,40,41].

Manoalide displays a preferential killing against oral cancer cells with little damage to normal oral cells. Similarly, betulinic acid (BA) selectively inhibits proliferation against a number of cancer cells but not on normal cells (peripheral blood lymphoblast) [42,43], and therefore BA was chosen as a positive control. Like manoalide, BA induces ROS generation, apoptosis, and proliferation, and these effects are suppressed by NAC treatment [34]. As shown in Figure 5B and Figure 6B, manoalide induces more MitoSOX generation and MitoMP depletion (JC-1 monomers generation) than that of BA, suggesting that manoalide is an effective oxidative stress inducer compared to BA in oral cancer cells. The anticancer effect of BA was independent on p53 mutant or wild types [42,43]. In the current study, all oral cancer cell lines harboring mutant p53 [44,45] and the role of p53 status warrants detailed investigation in future.

### 3.3. Manoalide Induced Oxidative stress Contributes to Apoptosis and DNA Damage Against Oral Cancer Cells

ROS-modulating drugs commonly induce apoptosis [29,40,41,46,47,48,49]. This is indicated by NAC and Z-VAD as apoptosis inhibitors. In our study, both show the suppressing effect on manoalide-induces subG1 accumulation and apoptosis (Figure 2B and Figure 3B), suggesting that oxidative stress plays a vital role in manoalide-induced apoptosis. Additionally, we found that Cas 8 and Cas 9 inhibitors suppressed the manoalide-induced c-Cas 3 activation using flow cytometry (Figure 3E). Accordingly, the role of extrinsic and intrinsic apoptosis may be involved in manoalide-induced apoptosis, and it warrants detailed investigation in future.

It is noted that apoptosis inhibitor Z-VAD cannot completely recover the manoalide-induced antiproliferation against oral cancer cells. Half and one-third of cell viabilities for Ca9-22 and CAL 27 cells were unable to be recovered (Figure 1D). These results suggest that apoptosis cannot completely attribute to antiproliferation effects of manoalide-treated oral cancer cells. Detailed studies of the involvement of other non-apoptosis mechanisms after manoalide treatment are warranted.

Moreover, oxidative stress is a high risk factor for inducing DNA damage [41,47]. Consistently, DNA double strand breaks (γH2AX) and oxidative DNA damage (8-oxodG) were induced in oral cancer cells upon manoalide exposure. Both γH2AX and 8-oxodG levels in manoalide-treated oral cancer cells were suppressed by NAC pretreatments (Figure 7B and Figure 8B). DNA damage also has a potential to induce apoptosis [50]. Accordingly, oxidative stress may induce DNA damage and lead to apoptosis. It is noted that the manoalide-induced γH2AX/8-oxodG expressions (Figure 7B and Figure 8B) and ROS production (Figure 4B) were also suppressed by Z-VAD pretreatments, suggesting that apoptosis may crosstalk to DNA damage in addition to oxidative stress.

It was reported that superoxide anion, such as MitoSOX, cannot cross the mitochondrial membrane [51]. However, we found that manoalide-induced MitoSOX generation and γH2AX/8-oxodG expressions (Figure 7C and Figure 8C) were suppressed by MitoTEMPO pretreatment. Since MitoTEMPO is the mitochondria-targeted superoxide inhibitor [52], the role of MitoSOX on DNA damage is explored by MitoTEMPO pretreatment. Our finding suggests that MitoSOX may directly or indirectly induce DNA double strand breaks and oxidative DNA damage. Accordingly, the validation and mechanism of MitoSOX-induced DNA damage warrants detailed investigation in future. Therefore, both intracellular ROS and mitochondrial superoxide (MitoSOX) may contribute to the manoalide-induced DNA damage in oral cancer cells.

The possible mechanism for preferential killing of manoalide against oral cancer cells but less damage to normal oral cells is discussed as follows. In HGF-1 cells, the ROS production is few (Figure 4A), leads to fewer annexin V-detected apoptosis (Figure 3A) and 8-oxodG DNA damage (Figure 8A) than that of oral cancer cells, and causes the oral cancer cell death but keeps normal oral cells alive.

### 3.4. Potential Target Molecules of Manoalide

Manoalide is known as an irreversible inhibitor for PLC [16] and PLA2 [53], as well as calcium channel blockers (CCBs) [17]. PLC inhibitors, such as U73122, were reported to induce apoptosis of human umbilical vein endothelial cells (HUVEC) [54]. PLA2 inhibitors, such as quercetin, were also summarized to inhibit inflammation and cancer proliferation [55]. CCBs, such as verapamil and diltiazem, have been reviewed for antiproliferation against several types of cancer cells in vitro and in vivo [56]. Accordingly, PLC, PLA2, and CCBs are the potential targets for manoalide. It warrants detailed investigation to explore the role of these potential targets between oral cancer and normal oral cells in future.

## 4. Materials and Methods

### 4.1. Cell and Drug Information

All human oral cancer cell lines (Ca9-22, CAL 27, HSC-3, OC-2, and SCC-9) and a normal oral cell line (HGF-1) were used from Health Science Research Resources Bank (HSRRB) (Osaka, Japan) and American Type Culture Collection (ATCC; Manassas, VA, USA) except for OECM1 [57], a generous gift from Dr. Wan-Chi Tsai (Kaohsiung Medical University, Taiwan). Cells were cultured in 5% CO_2_ at 37 °C with humidity and maintained by regular formula (Gibco, Grand Island, NY, USA) with 10% fetal bovine serum as previously described [49].

Manoalide (CAYMAN CHEMICAL, Ann Arbor, MI, USA) was dissolved in dimethyl sulfoxide (DMSO) for treatment. A ROS scavenger *N*-acetylcysteine (NAC) [58] (Sigma-Aldrich; St. Louis, MO, USA) was dissolved in double distilled water. The mitochondrial superoxide inhibitor MitoTEMPO [59] (Cayman Chemical, Ann Arbor, MI, USA), panapoptosis inhibitor Z-VAD-FMK [60], Cas 8 inhibitor Z-IETD-FMK, and Cas 9 inhibitor Z-LEHD-FMK (Selleckchem.com; Houston, TX, USA) was dissolved in DMSO. All experiments contain the same concentration of DMSO.

### 4.2. Cell Viability Assay

After drug treatment, the mitochondrial activity-based cell viability was determined by MTS assay (CellTiter 96 Aqueous One Solution, Promega, Madison, WI, USA) at 24 h [61], and the 3D microtissue spheroids viability of oral cancer cells was measured by the CellTiter-Glo^®^ 3D Cell Viability Assay (Promega, Madison, WI, USA) coupling with ATP level detection at 72 h [62].

### 4.3. Cell Cycle Assay

7AAD (Biotium, Inc., Hayward, CA, USA), a DNA dye, was applied to cell cycle analysis [63]. Briefly, drugs-treated cells were stained with 7AAD (1 μg/mL, 37 °C, 30 min). Finally, the cell cycle change was analyzed by Accuri™ C6 flow cytometry (Becton-Dickinson, Mansfield, MA, USA).

### 4.4. Annexin V/7AAD Assay for Apoptosis

Annexin V (Strong Biotech Corporation, Taipei, Taiwan) coupled with 7AAD was used for apoptosis analysis [64]. Briefly, drugs-treated cells were incubated with the mixture of annexin V-fluorescein isothiocyanate (FITC) (10 μg/mL) and 7AAD (1 μg/mL) at 37 °C for 30 min. Finally, the apoptosis expression was analyzed by Accuri™ C6 flow cytometry.

### 4.5. Western Blotting and c-Cas 3-Based Flow Cytometry for Apoptosis

Detailed steps of western blotting were previously described [60]. Briefly, the primary apoptosis antibodies (diluted 1:1000) including cleaved caspase-3 (c-Cas 3) rabbit mAb (Cell Signaling Technology, Inc., Danvers, MA, USA) were used. The internal control primary antibody (diluted 1:5000) was mAb-β-actin (Sigma-Aldrich, St. Louis, MO, USA). Following secondary antibody treatment, these protein signals were detected using enhanced chemiluminescence (ECL) substrate (WesternBright™ ECL HRP, Advansta, Menlo Park, CA, USA).

For c-Cas 3-based flow cytometry, cells were fixed with 70% ethanol, washed, and incubated with 1 μg/mL of c-Cas 3 (Asp175) rabbit mAb (Cell Signaling Technology) at 4 °C for overnight. After washing, cells were incubated with a secondary polyclonal antibody conjugated with Alexa Fluor 488 (ThermoFisher Scientific, San Jose, CA, USA) at room temperature for 1 h. Finally, the c-Cas 3 expression was analyzed by Accuri™ C6 flow cytometry. Cas 8 inhibitor Z-IETD-FMK (100 μM, 2 h) or Cas 9 inhibitor Z-LEHD-FMK (100 μM, 2 h) were applied to examine the involvement of Cas 8 and Cas 9 in apoptosis.

### 4.6. ROS Assay

DCFH-DA (Sigma-Aldrich; St. Louis, MO, USA) was used for ROS detecting dye [32]. Briefly, drugs-treated cells were incubated with DCFH-DA reagent (10 μM, 37 °C, 30 min). Finally, the ROS level was analyzed by Accuri™ C6 flow cytometry.

### 4.7. MitoSOX Assay

MitoSOX™ Red (Molecular Probes, Invitrogen, Eugene, OR, USA) was used as mitochondrial superoxide detecting dye [33]. Briefly, drugs-treated cells were incubated with MitoSOX reagent [48,49] (5 μM, 37 °C, 30 min). Finally, the MitoSOX level was analyzed by Accuri™ C6 flow cytometry.

### 4.8. MitoMP Assay

JC-1 (Merckmillipore) was used to detect mitochondrial membrane potential (MitoMP). JC-1 aggregate form generated red fluorescence indicating the normal function for MitoMP [35]. In contrast, JC-1 monomer form generated green fluorescence, indicating the dysfunction for MitoMP. Therefore, green fluorescent signals were counted as the decrease of MitoMP. Briefly, drugs-treated cells were treated with JC-1 (0.1 mM, 37 °C, 30 min). Finally, the MitoMP level was analyzed by Accuri™ C6 flow cytometry.

### 4.9. γH2AX Assay

DNA double strand break marker (γH2AX) was detected by antibody-based flow cytometry [40]. Briefly, drugs-treated cells were incubated with mouse primary antibody p-Histone H2A.X (Ser 139) (Santa Cruz Biotechnology, Santa Cruz, CA, USA) (1:100 dilution, 4 °C, 1 h) and washed for incubation with the secondary antibody-labeled with Alexa Fluor 488 (Cell Signaling Technology) (1:10000 dilution, 4 °C, 1 h). Finally, the γH2AX level was analyzed by Accuri™ C6 flow cytometry.

### 4.10. 8-oxodG Assay

8-oxodG was detected by antibody-based flow cytometry using a fluorometric OxyDNA assay kit (#500095; EMD Millipore, Darmstadt, Germany) [65,66]. Briefly, drugs-treated cells were incubated in antibody-labeled with FITC (10× dilution, 4 °C, 1 h). Finally, the 8-oxodG level was analyzed by Accuri™ C6 flow cytometry.

### 4.11. Statistical Analysis

Using JMP^®^ 12 software, the significance of multiple comparisons between different treatments were analyzed by one-way ANOVA with Tukey HSD Post Hoc Test. Data showing no overlapping same small letters represent significant difference.

## 5. Conclusions

**Figure 9:**
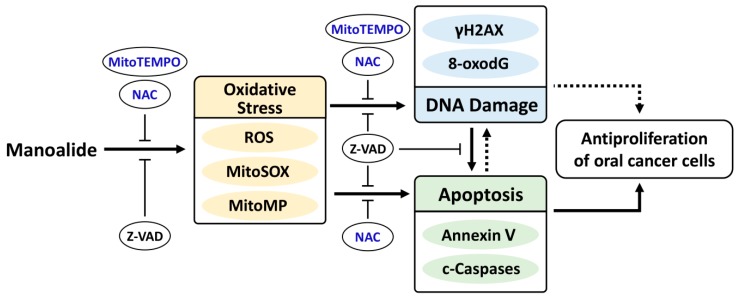
Expected mechanism of manoalide-induced preferential antiproliferation on oral cancer cells. Manoalide may preferentially kill oral cancer cells by inducing oxidative stress, such as ROS and MitoSOX productions, as well as MitoMP depolarization. These oxidative stresses may induce DNA double strand break damage and oxidative DNA 8-oxodG DNA damage. DNA damage may induce apoptosis and inhibit proliferation [67]. Finally, these oxidative stress and DNA damage changes cooperate to induce apoptosis [50] and lead to preferential antiproliferation of oral cancer cells. Additionally, apoptosis may also regulate DNA damage and oxidative stress. Solid lines (arrow and T) indicate the activating and inhibiting results from this study, whereas dashed lines indicate the mechanism is supported by literature.

This study confirmed the hypothesis that manoalide may preferentially inhibit the proliferation of oral cancer cells. We found several types of results supporting that oxidative stresses were induced by manoalide. The oxidative stresses, such as intracellular ROS and MitoSOX/MitoMP, were also involved in manoalide-induced apoptosis and DNA damages in oral cancer cells. Finally, these mechanisms may contribute to preferentially inhibit the proliferation of oral cancer cells (Figure 9). Taken together, this study firstly shows that manoalide preferentially kills oral cancer cells without cytotoxic side effects to normal oral cells. 

## Supplementary Materials

The following are available online at https://www.mdpi.com/2072-6694/11/9/1303/s1, Figure S1: 3D spheroid formation and cell morphology of oral cancer cells after manoalide treatment and its NAC/Z-VAD effects, Figure S2: Cell cycle changes of manoalide-treated oral cancer (Ca9-22 and CAL 27) cells, Figure S3: Apoptosis changes in manoalide-treated oral cancer (Ca9-22 and CAL 27) cells and normal oral (HGF-1) cells, Figure S4: Western blotting for procaspase 3 and cleaved caspase 3 (c-Cas 3) in manoalide-treated oral cancer (Ca9-22 and CAL 27) cells, Figure S5: Raw data for western blotting of cleaved caspase 3 (c-Cas 3) with or without NAC and Z-VAD pretreatment in manoalide-treated oral cancer (Ca9-22 and CAL 27) cells, Figure S6: Fluorescence staining of cleaved caspases 8 and 9 (c-Cas 8/9) and the effects of Cas 8/9 inhibitors on c-Cas 3-based flow cytometry, Figure S7: ROS changes in manoalide-treated oral cancer (Ca9-22 and CAL 27) and normal oral (HGF-1) cells, Figure S8: Change of MitoSOX production in manoalide-treated oral cancer (Ca9-22 and CAL 27) cells, Figure S9: Change of MitoMP in manoalide-treated oral cancer (Ca9-22 and CAL 27) cells, Figure S10: Change of γH2AX DNA damage in manoalide-treated oral cancer (Ca9-22 and CAL 27) cells, Figure S11: Change of 8-oxodG DNA damage in manoalide-treated oral cancer (Ca9-22 and CAL 27) cells, and Figure S12: ABTS and hydroxyl scavenging assays for manoalide.
