# Supplementary material for: Manoalide Preferentially Provides Antiproliferation of Oral Cancer Cells by Oxidative Stress-Mediated Apoptosis and DNA Damage
Source: Cancers (Basel). 2019 Sep 4;11(9):1303. doi: 10.3390/cancers11091303 (PMC6770486; doi:10.3390/cancers11091303)
Supplement: Supplementary file 1 [file cancers-11-01303-s001.pdf]

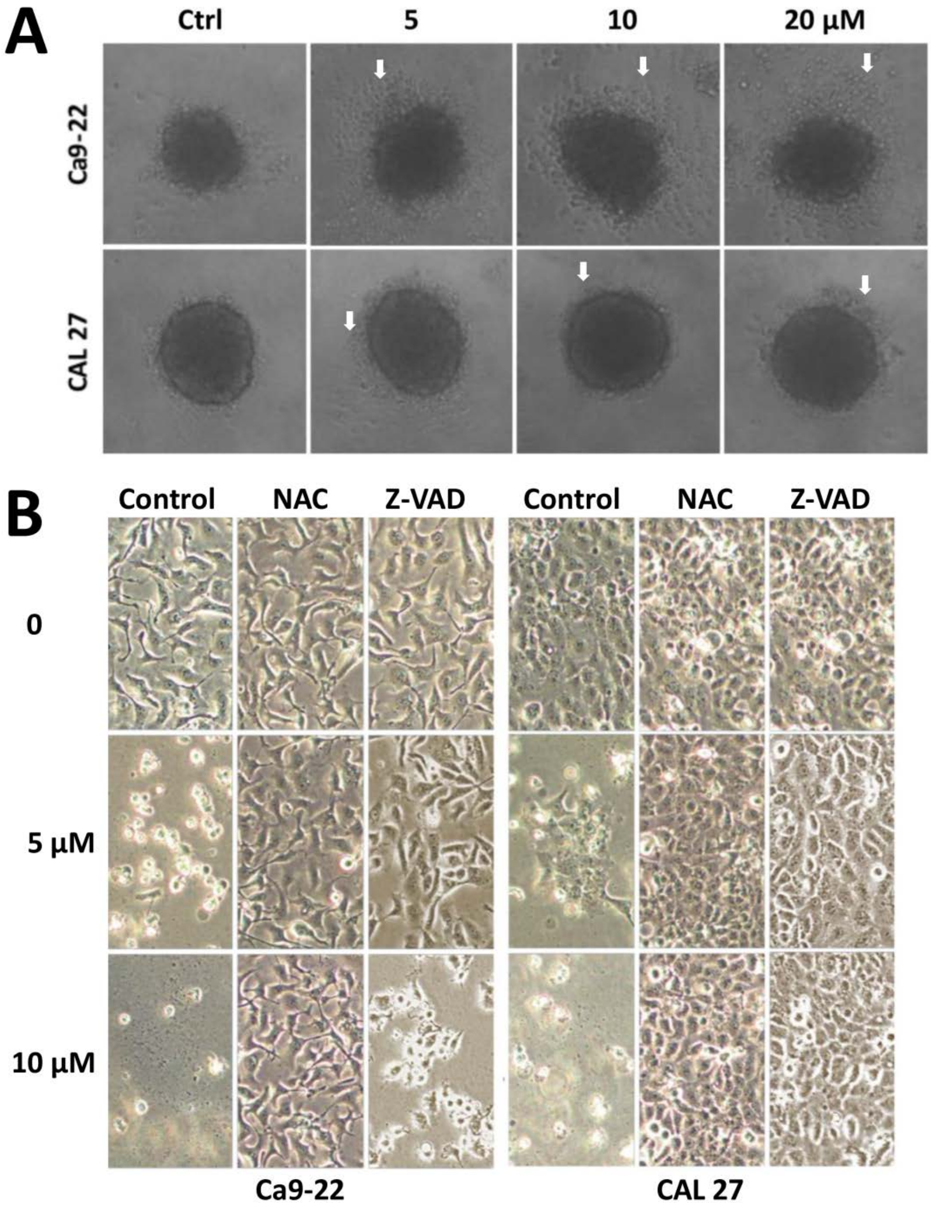

Figure S1. 3D spheroid formation and cell morphology of oral cancer cells after manoalide treatment and its NAC/Z-VAD effects. (**A**) Photo images of 3D spheroid formation for manoalide-treated oral cancer (Ca9-22 and CAL 27) cells for 72 h. Cells were treated with 0, 5, 10, and 20 μM of manoalide. The white arrow indicates the dead cells spreading from 3D spheroid. However, the quantitative assay to determine the cell death is analyzed by ATP assay (Figure 1C). (**B**) NAC and Z-VAD effects on cell morphology of manoalide-treated oral cancer cells. Pretreatment conditions were 8 mM, 1 h for NAC and 100 μM, 2 h for Z-VAD. Following pretreatment or not, oral cancer (Ca9-22 and CAL 27) cells were post-incubated with 5 and 10 μM manoalide for 24 h. Cell morphology was photographed at 100x magnification. The statistical results of Figures S1A and S1B are shown in Figures 1C and 1D, respectively.

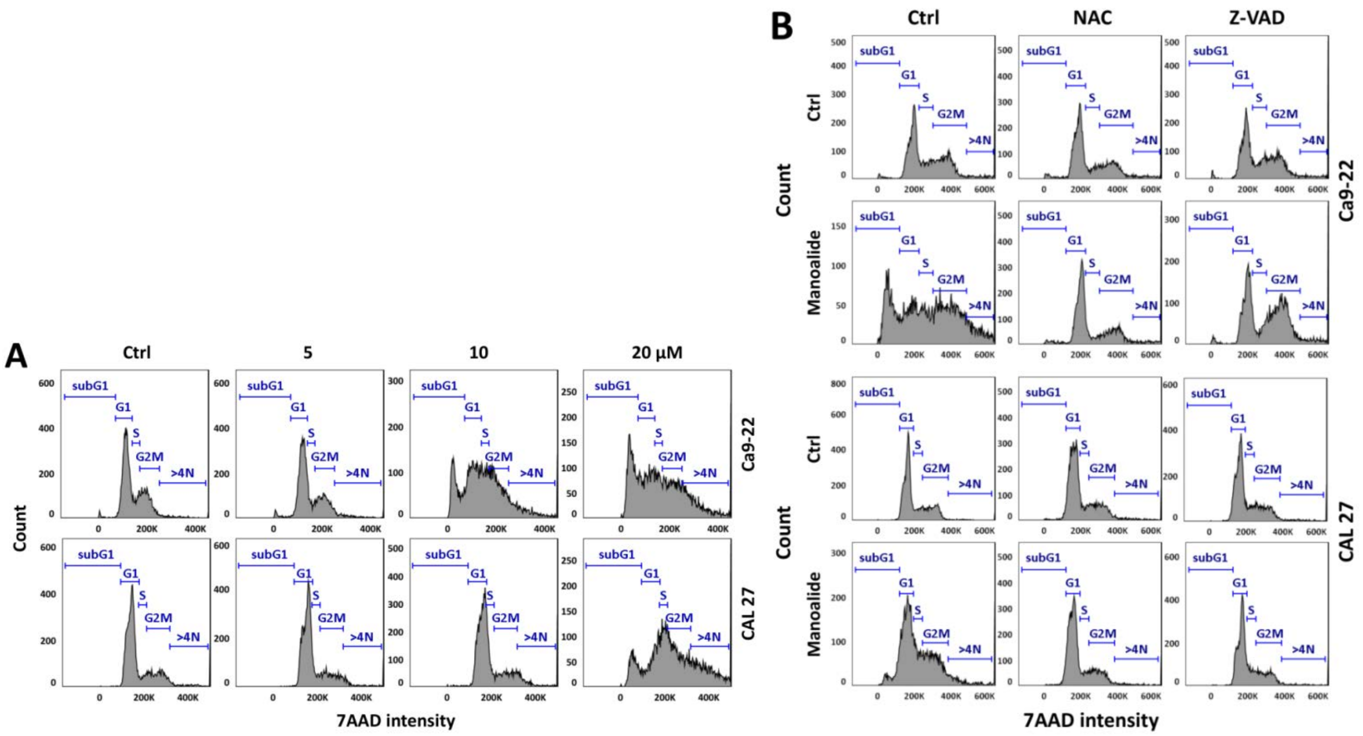

Figure S2. Cell cycle changes of manoalide-treated oral cancer (Ca9-22 and CAL 27) cells. (**A**) Typical cell cycle patterns of manoalide-treated oral cancer cells. Cells were treated with 0, 5, 10, and 20 μM of manoalide for 24 h. (**B**) Typical cell cycle patterns in NAC, Z-VAD, and/or manoalide-treated oral cancer cells. Cells were pretreated with 8 mM, 1 h for NAC or 100 μM, 2 h for Z-VAD and post-incubated with 10 μM of manoalide for 24 h. The statistical results of Figures S2A and S2B are shown in Figures 2A and 2B, respectively.

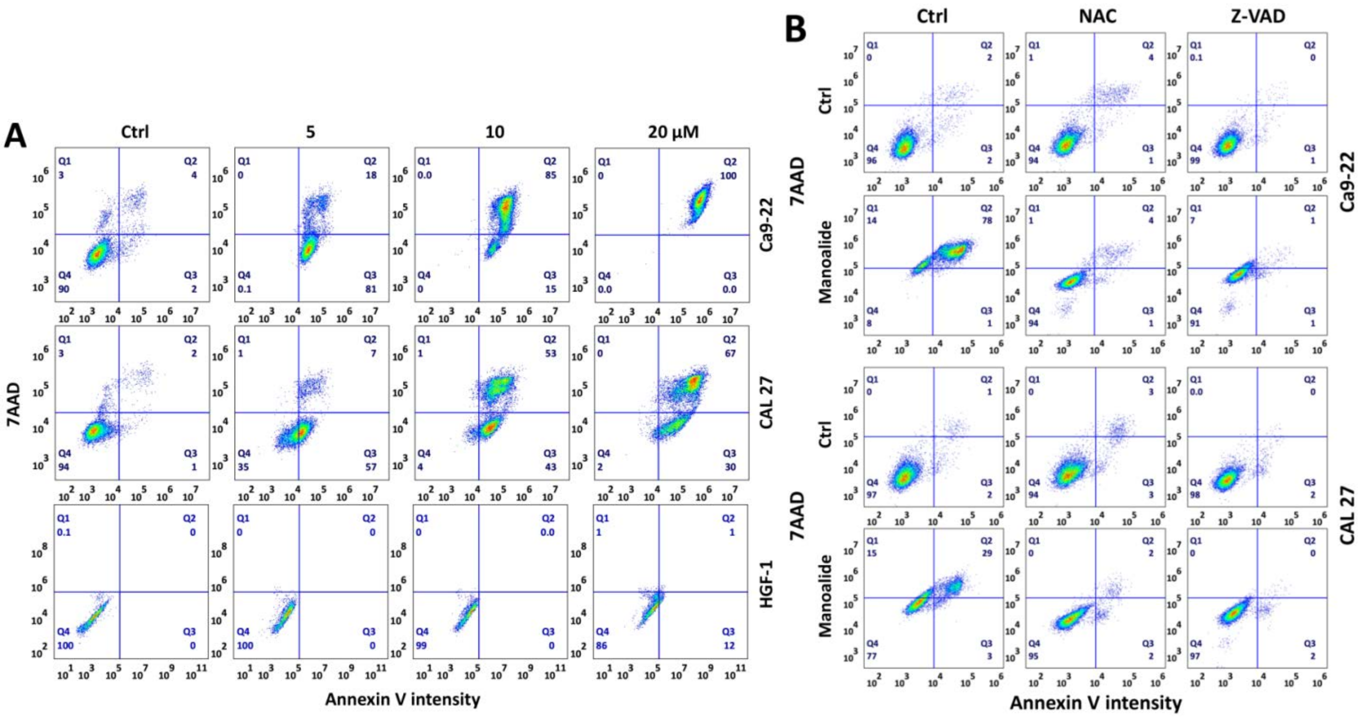

Figure S3. Apoptosis changes in manoalide-treated oral cancer (Ca9-22 and CAL 27) cells and normal oral (HGF-1) cells. (**A**) Typical pattern of the annexin V/7AAD method in manoalide-treated oral cancer and normal oral cells. Cells were treated with different concentrations of manoalide for 24 h. Early and late apoptosis were respectively counted by the populations in the annexin V (+)/7AAD (-) and annexin V (+)/7AAD (+) regions, i.e., Q3 and Q2. (**B**) Typical pattern of annexin V/7AAD method in NAC, Z-VAD, and/or manoalide-treated oral cells. Cells were pretreated with NAC (8 mM, 1 h) or Z-VAD (100 μM, 2 h), and posttreated with manoalide (10 μM, 24 h). Apoptosis was represented by the sum of early and late apoptosis, i.e., annexin V (+)/7AAD (+ or -). The statistical results of Figures S3A and S3B are shown in Figures 3A and 3B, respectively.

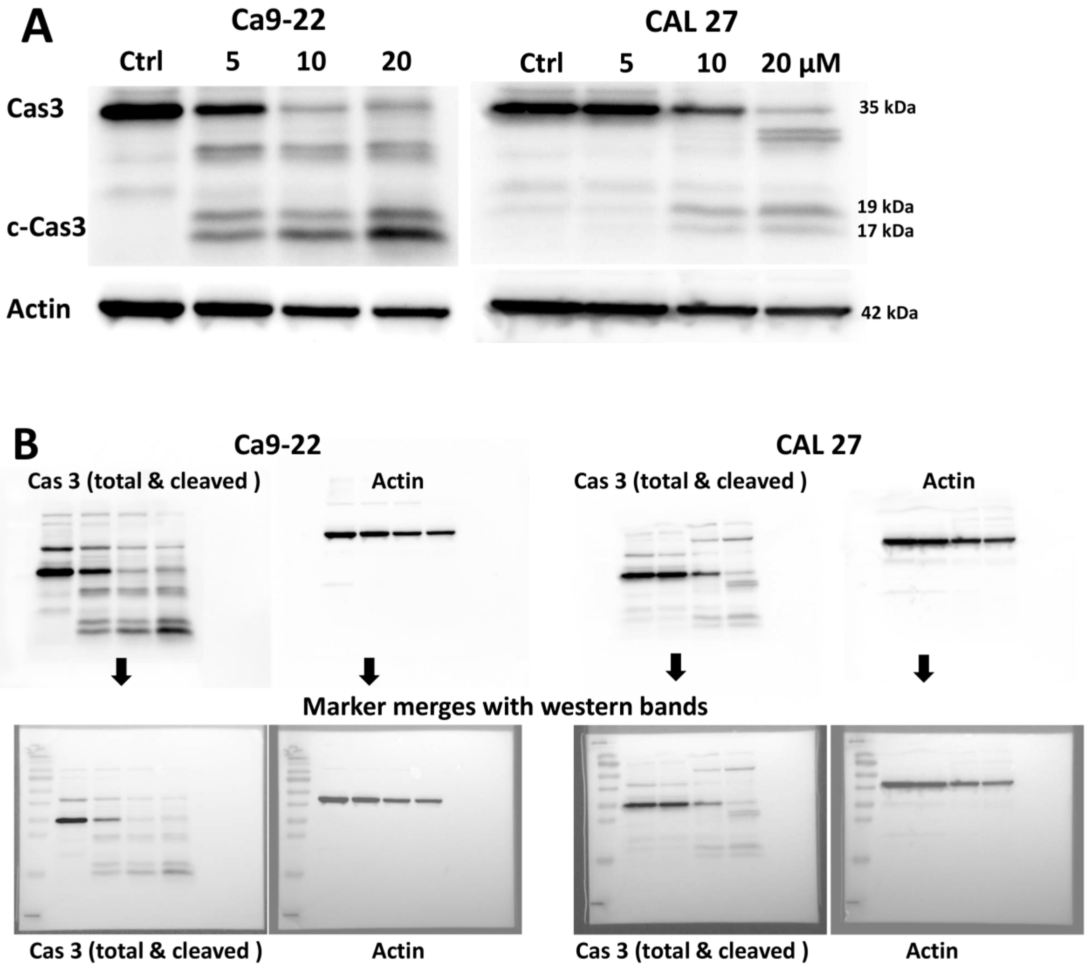

Figure S4. Western blotting for procaspase 3 and cleaved caspase 3 (c-Cas 3) in manoalide-treated oral cancer (Ca9-22 and CAL 27) cells. Cells were treated with different concentrations of manoalide for 24 h. (**A**) Western blotting for procaspase 3 and c-Cas 3. (**B**) Raw data for Figure S4A.

A

Figure 2C (Left side)

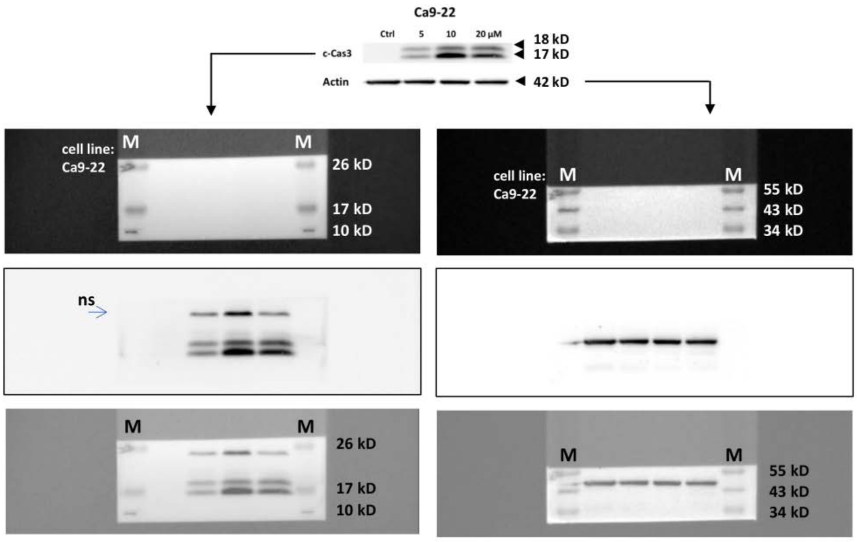

Marker (M) image

ECL image

Marker/ECL merge image (50% transparency)

ns = nonspecific

Figure 2C (Right side)

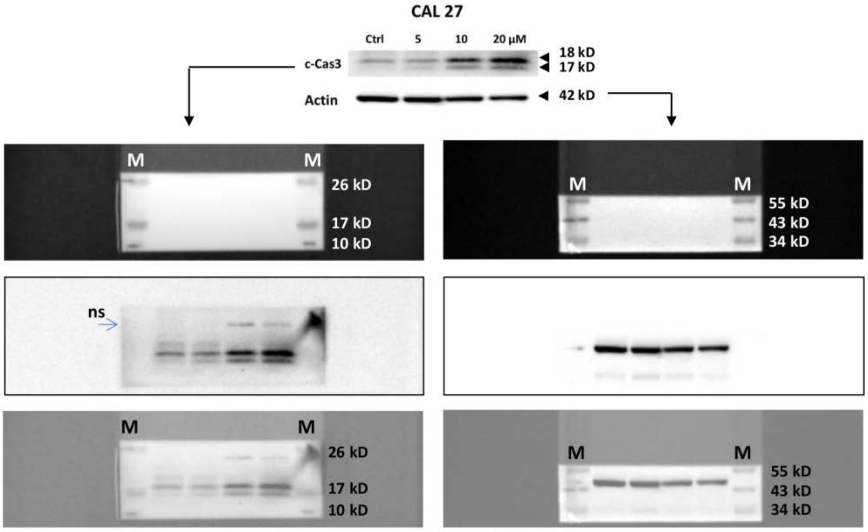

Marker (M) image

ECL image

Marker/ECL merge image (50% transparency)

ns = nonspecific

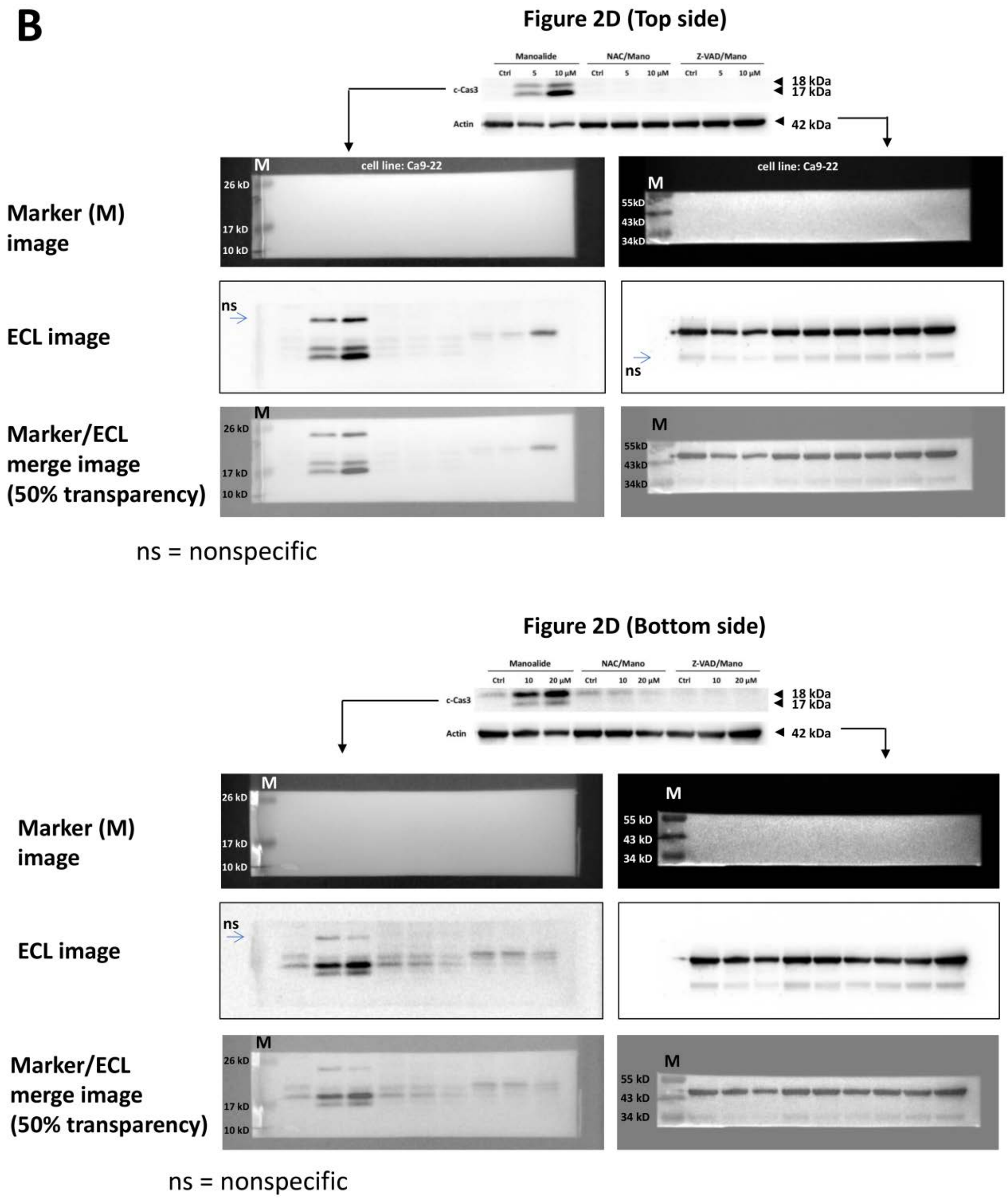

Figure S5. Raw data for western blotting of cleaved caspase 3 (c-Cas 3) with or without NAC and Z-VAD pretreatment in manoalide-treated oral cancer (Ca9-22 and CAL 27) cells. (**A**) Raw data for western blotting (Figure 3C) of c-Cas 3 in different concentrations of manoalide-treated oral cancer cells. (**B**) Raw data for western blotting (Figure 3D) of c-Cas 3 in different concentrations of manoalide-treated oral cancer cells with NAC or Z-VAD pretreatment.

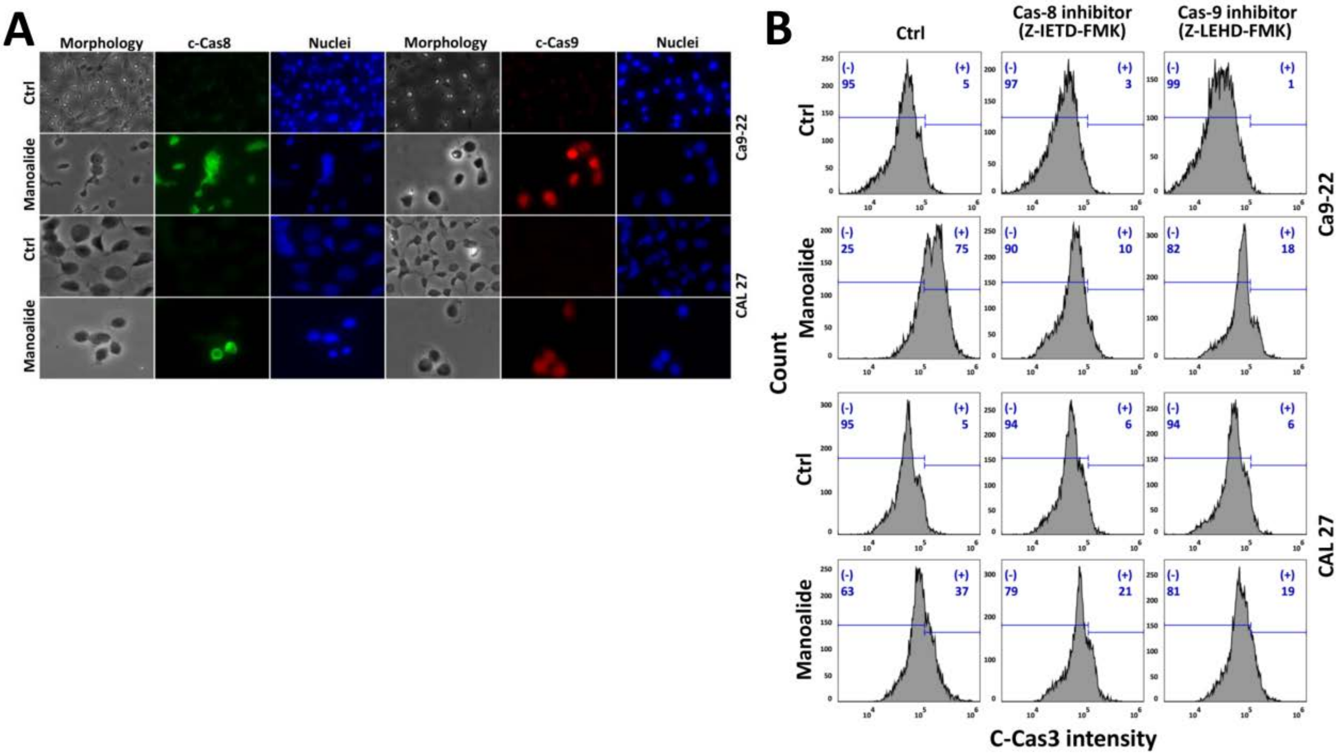

Figure S6. Fluorescence staining of cleaved caspases 8 and 9 (c-Cas 8/9) and the effects of Cas 8/9 inhibitors on c-Cas 3-based flow cytometry. (**A**) Typical fluorescence pattern of c-Cas 8/9 in manoalide-treated oral cancer (Ca9-22 and CAL 27) cells. Cells were treated with manoalide (10 μM) for 12 h. Primary antibodies against c-Cas 8 (Asp391) (18C8) rabbit monoclonal antibody (mAb) [1:1000] and c-Cas 9 (Asp330) (D2D4) rabbit mAb [1:1000] (Cell Signaling Technology, Inc., Danvers, MA, USA) were used and the matched secondary antibodies (Alexa Fluor 488 or 568 conjugated polyclonal goat anti-rabbit antibody (ThermoFisher Scientific, San Jose, CA, USA) [1: 500] were used respectively. (**B**) Typical pattern of c-Cas 3-based flow cytometry in Cas 8 inhibitor, Cas 9 inhibitor, and/or manoalide-treated oral cells. Cells were pretreated with Cas 8 inhibitor Z-IETD-FMK (100 μM, 2 h) or Cas 9 inhibitor Z-LEHD-FMK (100 μM, 2 h), and posttreated with manoalide (10 μM, 24 h). The statistical result of Figure S6B is shown in Figure 3E.

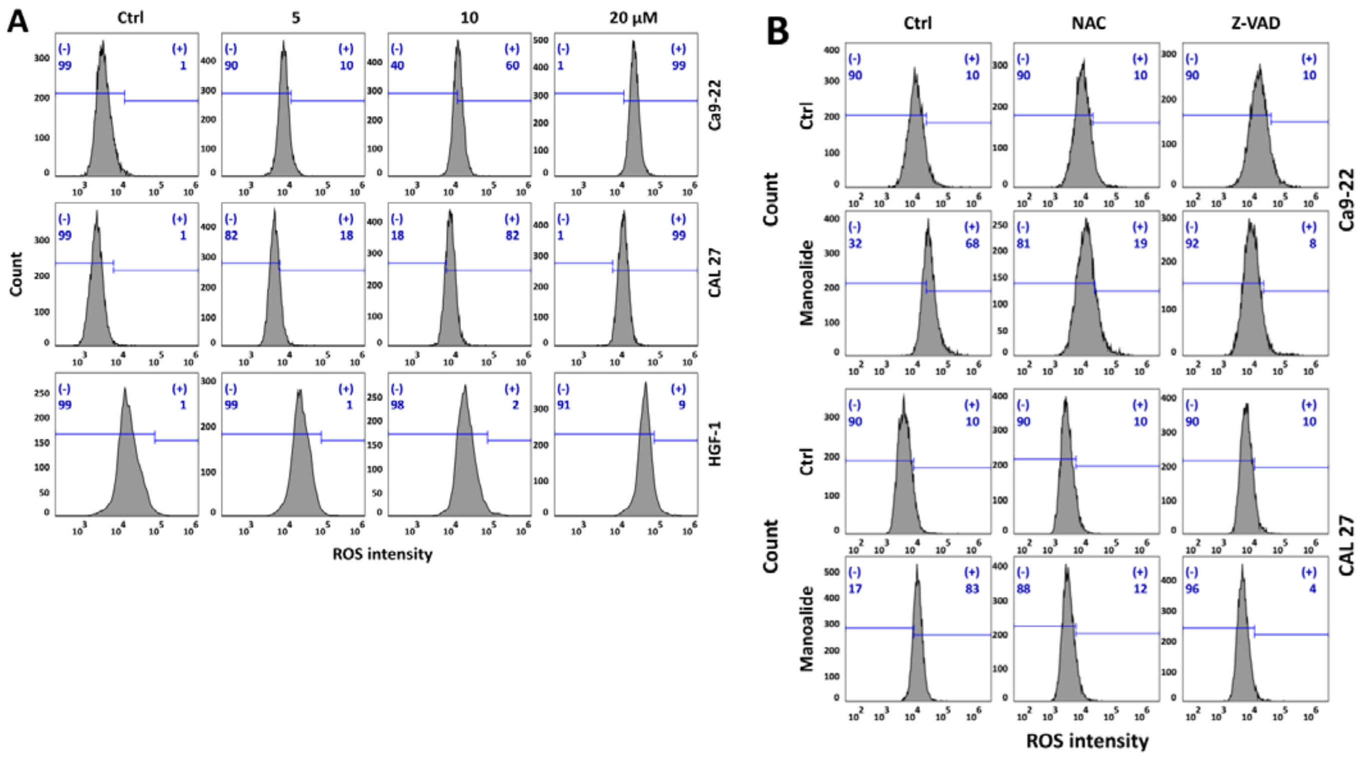

Figure S7. ROS changes in manoalide-treated oral cancer (Ca9-22 and CAL 27) and normal oral (HGF-1) cells. Cells were treated with different concentrations of manoalide for 10 min. (**A**). Typical ROS patterns of manoalide-treated oral cancer cells. Right ranges indicate the ROS-positive population. (**B**) Typical ROS patterns in NAC, Z-VAD, and/or manoalide-treated oral cells. Cells were pretreated with NAC (8 mM, 1 h) or Z-VAD (100 μM, 2 h), and posttreated with manoalide (10 μM, 10 min). Right ranges indicate the ROS-positive population. The statistical results of Figures S7A and S7B are shown in Figures 4A and 4B, respectively.

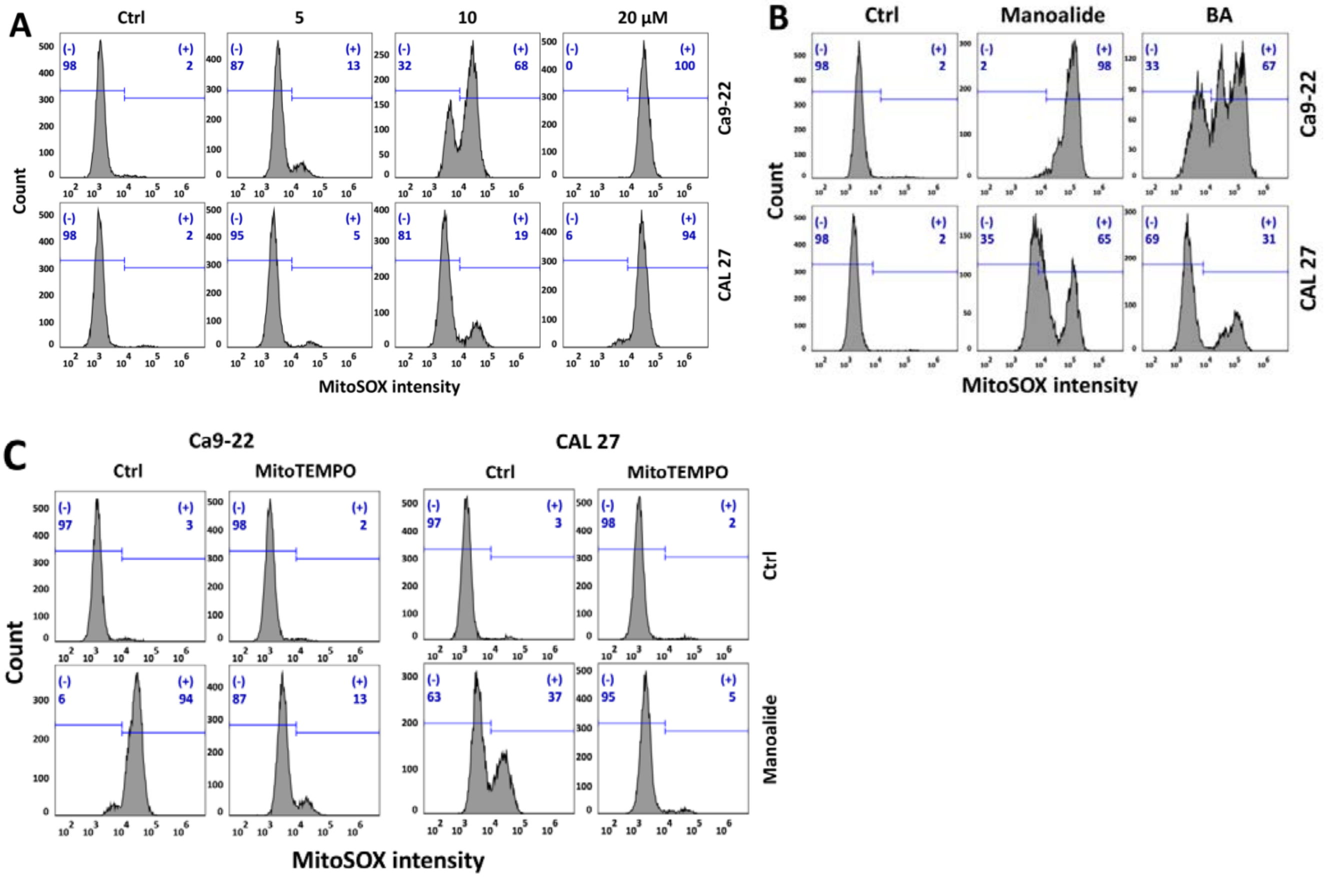

Figure S8. Change of MitoSOX production in manoalide-treated oral cancer (Ca9-22 and CAL 27) cells. (**A**) Typical MitoSOX patterns for manoalide-treated oral cancer cells using flow cytometry. Cells were treated with different concentrations of manoalide for 24 h. (**B**) Typical positive control of MitoSOX patterns for oral cancer cells. Cells were treated with betulinic acid (BA; 25 µM, 24 h) as the positive control treatment for comparison to manoalide (10 µM, 24 h). (**C**) Typical MitoSOX patterns in MitoTEMPO and/or manoalide-treated oral cells. Cells were pretreated with MitoTEMPO (20 µM, 1 h) and posttreated with manoalide (10 µM, 24 h). The statistical results of Figures S8A, S8B, and S8C are shown in Figures 5A, 5B, and 5C, respectively.

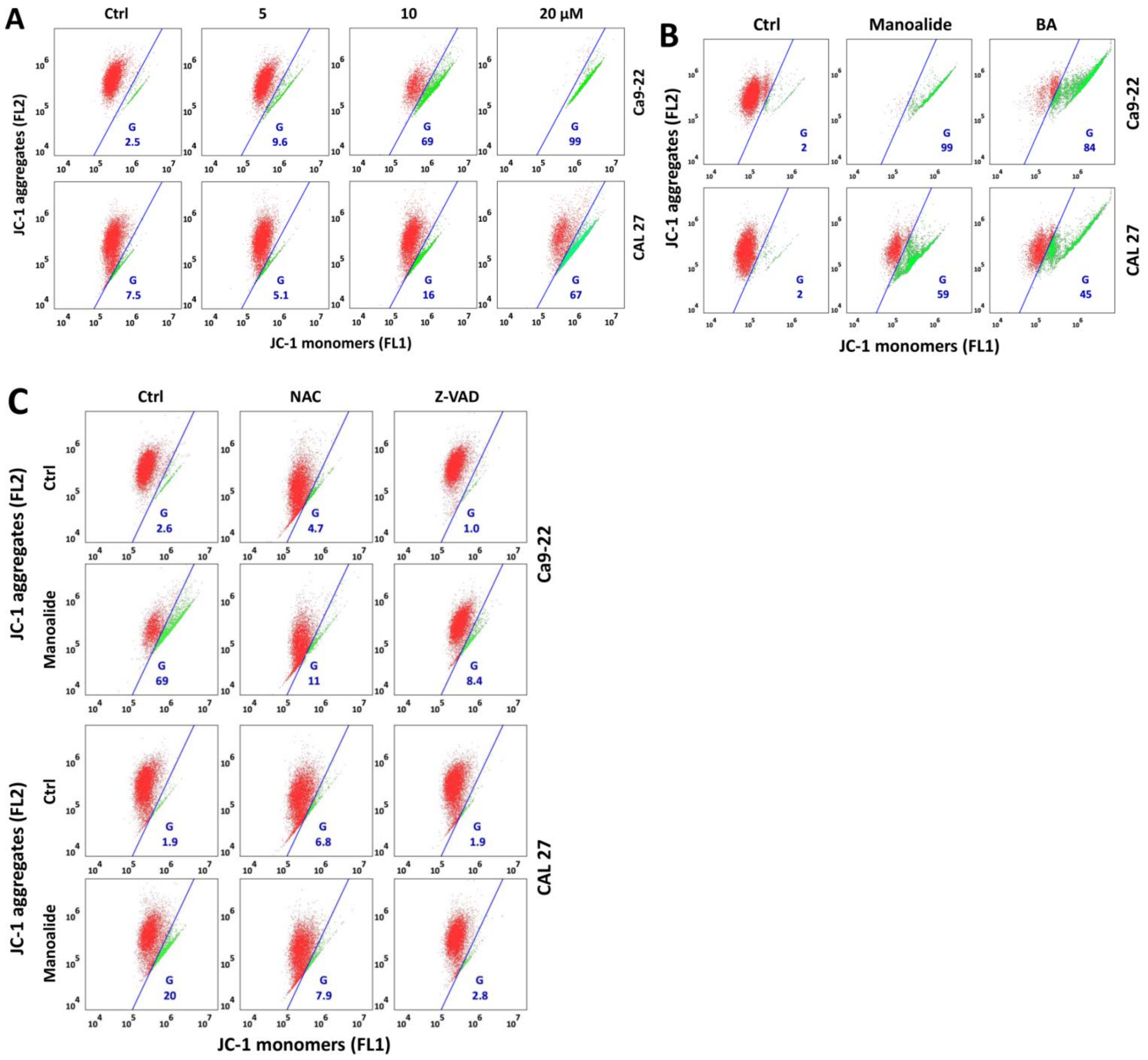

Figure S9. Change of MitoMP in manoalide-treated oral cancer (Ca9-22 and CAL 27) cells. For MitoMP pattern, slash separates populations with high (red, FL2) and low (green, FL1) MitoMP. (**A**) Typical MitoMP patterns for manoalide-treated oral cancer cells using flow cytometry. Cells were treated with different concentrations of manoalide for 24 h. The increase of low MitoMP (green) indicates the MitoMP depolarization. (**B**) Typical positive control of low MitoMP patterns for oral cancer cells. Cells were treated with betulinic acid (BA; 25 μM, 24 h) as the positive control treatment for comparison to manoalide (10 μM, 24 h). (**C**) Typical MitoMP patterns of NAC, Z-VAD, and/or manoalide-treated oral cells. Cells were pretreated with NAC (8 mM, 1 h) or Z-VAD (100 μM, 2 h) and posttreated with manoalide (10 μM, 24 h). The statistical results of Figures S9A, S9B, and S9C are shown in Figures 6A, 6B, and 6C, respectively.

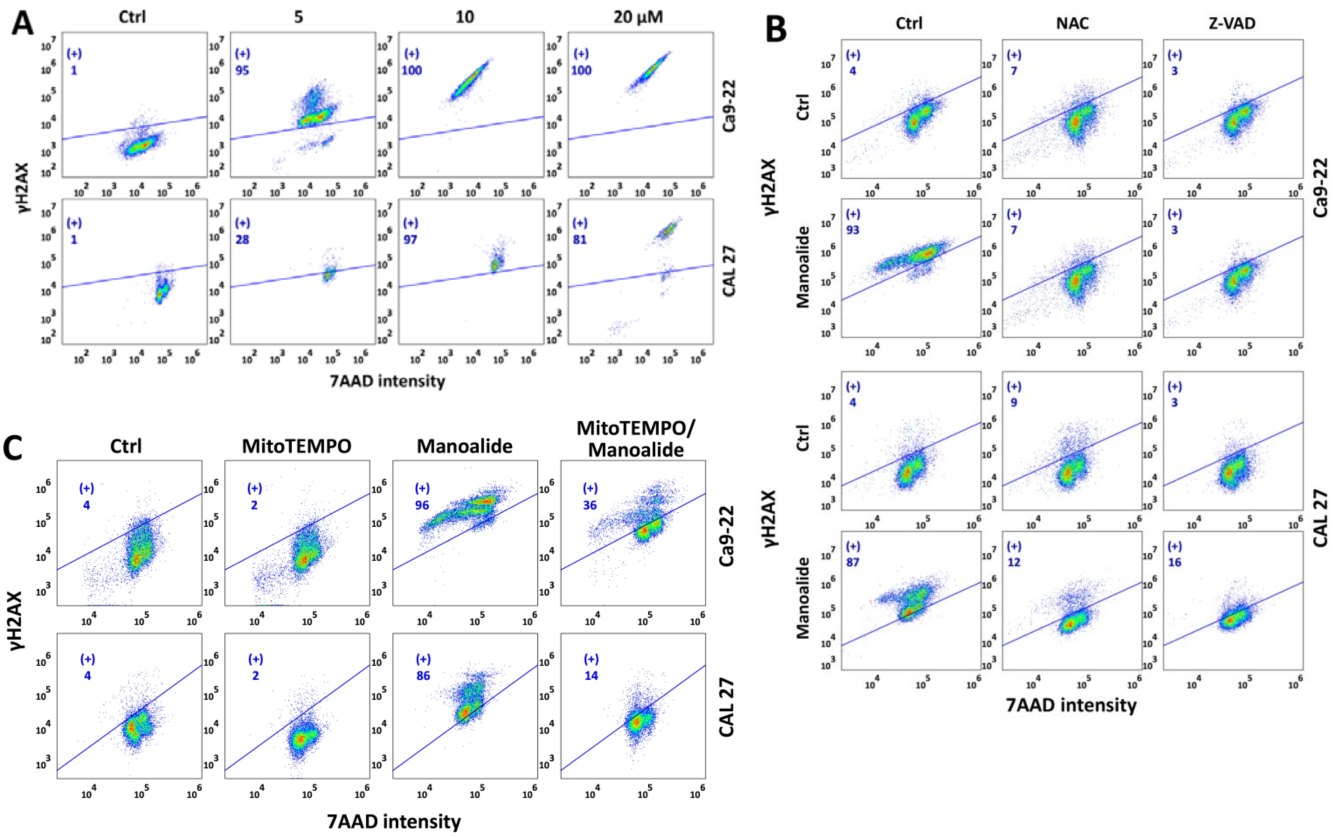

Figure S10. Change of γH2AX DNA damage in manoalide-treated oral cancer (Ca9-22 and CAL 27) cells. Cells were treated with the indicated concentrations of manoalide for 24 h. (**A**) Typical γH2AX pattern of manoalide-treated oral cancer cells. A slash separates populations with positive (up) and negative (down) γH2AX populations. (**B**) Typical γH2AX pattern patterns and statistical results in NAC, Z-VAD, and/or manoalide-treated oral cancer cells. Cells were pretreated with 8 mM, 1 h for NAC or 100 μM, 2 h for Z-VAD and post-incubated with 10 μM of manoalide for 24 h. (**C**) Typical γH2AX pattern of γH2AX (+) (%) in MitoSOX inhibitor (MitoTEMPO) and/or manoalide-treated oral cancer cells. Cells were pretreated with MitoTEMPO (20 μM, 1 h) and posttreated with manoalide (10 μM, 24 h). The statistical results of Figures S10A, S10B, and S10C are shown in Figures 7A, 7B, and 7C, respectively.

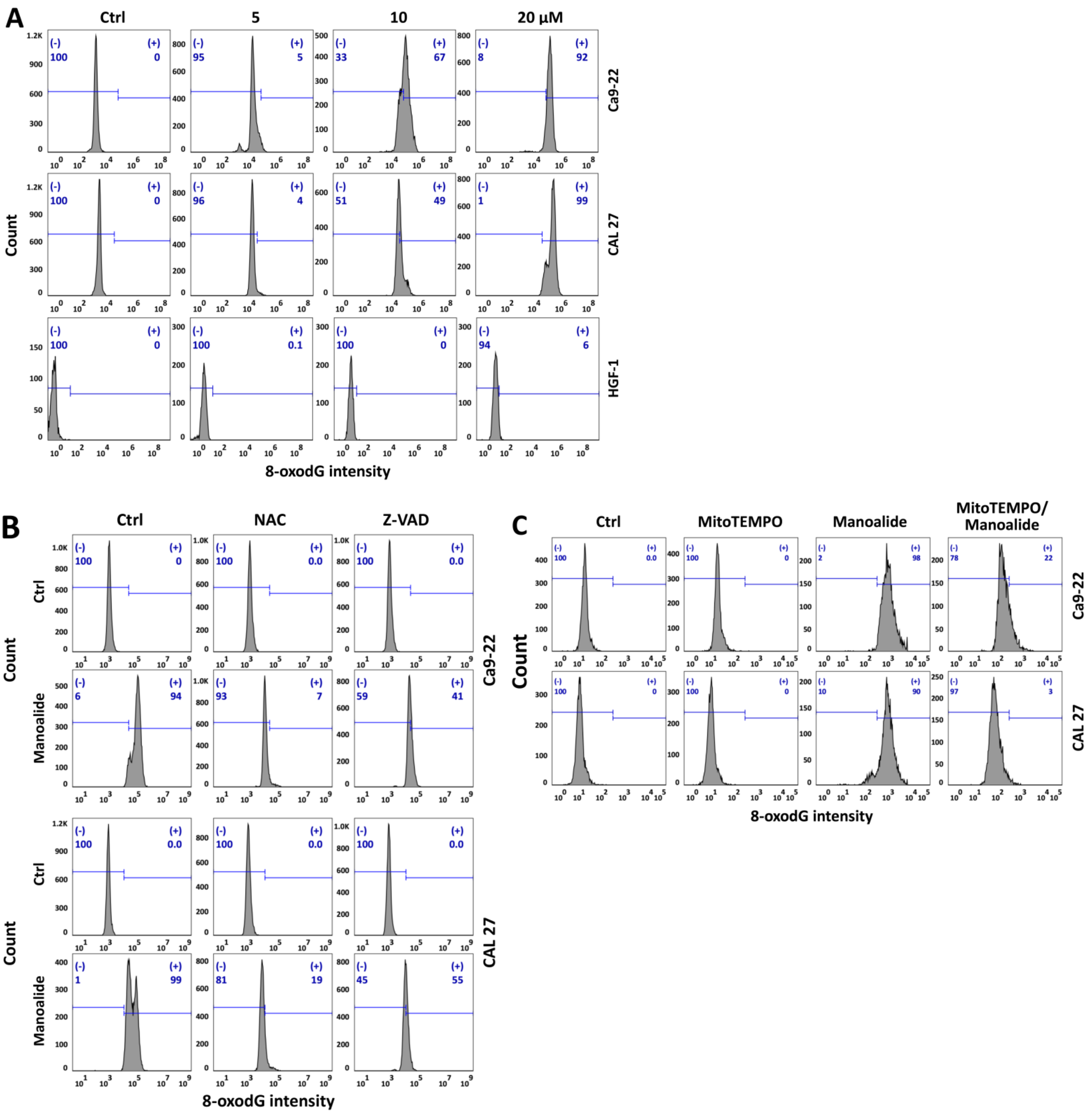

Figure S11. Change of 8-oxodG DNA damage in manoalide-treated oral cancer (Ca9-22 and CAL 27) and normal oral (HGF-1) cells. Cells were treated with the indicated concentrations of manoalide for 24 h. (**A**) Typical 8-oxodG patterns of manoalide-treated oral cancer and normal oral cells. (+) indicates the 8-oxodG-positive population. (**B**) Typical 8-oxodG patterns in NAC, Z-VAD, and/or manoalide-treated oral cancer cells. Cells were pretreated with 8 mM, 1 h for NAC or 100 μM, 2 h for Z-VAD and post-incubated with 10 μM of manoalide for 24 h. (**C**) Typical 8-oxodG pattern of 8-oxodG (+) (%) in MitoSOX inhibitor (MitoTEMPO) and/or manoalide-treated oral cancer cells. Cells were pretreated with MitoTEMPO (20 μM, 1 h) and posttreated with manoalide (10 μM, 24 h). The statistical results of Figure S11A, S11B, and S11C are shown in Figures 8A, 8B, and 8C, respectively.

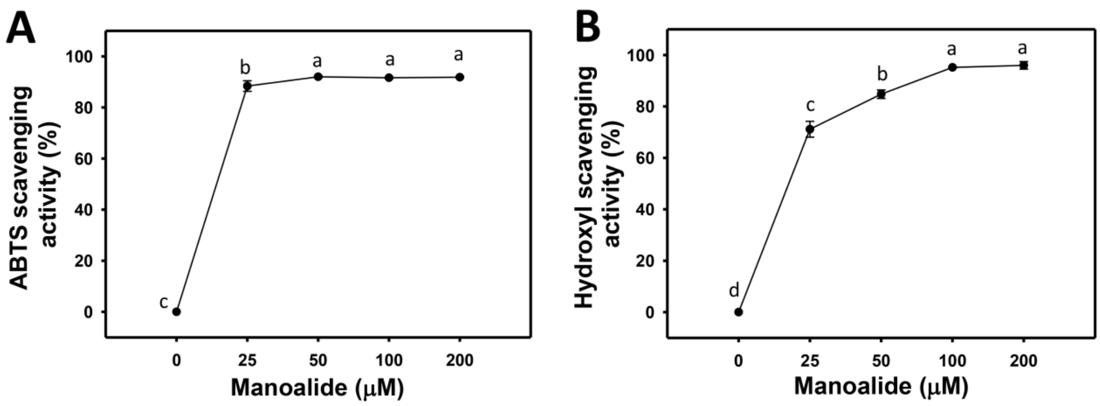

Figure S12: ABTS and hydroxyl scavenging assays for manoalide. (**A**) ABTS scavenging activity of manoalide (0, 25, 50, 100, and 200 μM). (**B**) Hydroxyl scavenging activity of manoalide. Data were analyzed by one-way ANOVA with Tukey HSD Post Hoc Test. Data, means ± SDs (n = 3). Data showing no overlapping same small letters represent significant difference (p < 0.05–0.001).
